# Association between metabolic syndrome and cataract: a meta-analysis

**DOI:** 10.1038/s41433-025-03910-2

**Published:** 2025-07-11

**Authors:** Caijuan Liu, Yinbo Zhang, Xiaowen Cao, Zhimin Chen

**Affiliations:** 1 Department of cataract, Hebei Eye Hospital, Hebei Provincial Key Laboratory of Ophthalmology, Hebei Provincial Clinical Research Center for Eye Diseases, Xingtai, China; 2 Shijiazhuang Medical Department, Hebei Eye Hospital, Hebei Provincial Key Laboratory of Ophthalmology, Hebei Provincial Clinical Research Center for Eye Diseases, Shijiazhuang, China

**Keywords:** Eye abnormalities, Metabolic disorders

## Abstract

**Background:**

Metabolic syndrome (MetS) has been suggested to be associated with an increased risk of cataract in adults. However, the evidence remains inconclusive. This meta-analysis aimed to clarify this potential association.

**Methods:**

We conducted a systematic search of PubMed, Embase, and Web of Science up to July 31, 2024, for observational studies evaluating the association between MetS and cataract. Data were pooled using a random-effects model to calculate risk ratios (RR) with 95% confidence intervals (CI). Heterogeneity was assessed with the Cochrane Q test and I² statistics. Subgroup analyses were performed based on study characteristics.

**Results:**

Ten studies with 379,464 participants were included. The meta-analysis showed that MetS was significantly associated with an increased risk of cataract (RR: 1.28, 95% CI: 1.16–1.41, *p* < 0.001; I² = 90%). Subgroup analyses indicated that the association was stronger in participants aged ≥57 years compared to those <57 years (*p* for subgroup difference <0.001) and in studies using the International Diabetes Federation criteria for MetS diagnosis compared to the National Cholesterol Education Program criteria (p for subgroup difference <0.001). No significant differences were found by geographic region, sex, or cataract types. Sensitivity analyses confirmed the robustness of these findings. Egger’s regression test showed no significant publication bias (*p* = 0.44).

**Conclusion:**

MetS is associated with an increased risk of cataract in adults, particularly in older populations. Further studies are needed to explore the underlying mechanisms and develop prevention strategies.

## Introduction

Cataract, characterized by the clouding of the eye’s crystalline lens, is one of the leading causes of visual impairment and blindness worldwide, affecting millions of individuals, particularly the elderly [1–3]. The global prevalence of cataract is rising due to population aging, posing a substantial public health burden and highlighting the need for effective prevention strategies [4, 5]. While aging remains the most significant risk factor, accumulating evidence suggests that metabolic disorders, such as metabolic syndrome (MetS), may also contribute to cataract development [6, 7].

By definition, MetS is a cluster of interconnected metabolic abnormalities, including central obesity, hypertension, dyslipidaemia, hyperglycaemia, and insulin resistance, which collectively increase the risk of cardiovascular disease and type 2 diabetes mellitus [8–10]. The concept of metabolic syndrome (MetS) was first introduced by the World Health Organization (WHO) in 1998, which emphasized insulin resistance as a core component along with other metabolic abnormalities [11]. Since then, several other definitions have been developed, among which two widely accepted ones are from the International Diabetes Federation (IDF) and the National Cholesterol Education Program Adult Treatment Panel III (NCEP-ATP III). The IDF defines MetS as central obesity plus any two of the following: elevated triglycerides, low high-density lipoprotein cholesterol (HDL-C), high blood pressure, or raised fasting plasma glucose [12]; while the NCEP-ATP III criteria require three or more of the above-mentioned metabolic abnormalities, regardless of central obesity [13]. The prevalence of MetS is increasing globally, even in children and adolescents, driven by sedentary lifestyles and dietary changes, and has become a significant public health concern [14].

Several pathological mechanisms have been proposed to explain the potential link between MetS and cataract formation. Hyperglycaemia and insulin resistance, key components of MetS, can lead to osmotic stress and oxidative damage in the lens, promoting cataractogenesis [15]. Dyslipidaemia and hypertension are also associated with increased oxidative stress, inflammatory responses, and endothelial dysfunction [16, 17], which may further contribute to lens opacity. Despite these plausible mechanisms, findings from previous observational studies investigating the association between MetS and cataract risk have been inconsistent [18–27], likely due to differences in study design, population characteristics, diagnostic criteria, and confounding factors. Given these inconclusive findings, we conducted a comprehensive meta-analysis of observational studies to evaluate the association between MetS and the risk of cataract in adults.

## Methods

The authors adhered to PRISMA 2020 guidelines [28, 29] and the Cochrane Handbook for Systematic Reviews and Meta-analyses [30] in conducting this meta-analysis, covering study design, data collection, statistical analysis, and result interpretation. The protocol of the study has been registered in PROSPERO with the identifier CRD42024594117.

### Literature search

To locate studies pertinent to this meta-analysis, we queried PubMed, Embase, and Web of Science with an extensive array of search terms, which included: (1) “metabolic syndrome” OR “insulin resistance syndrome” OR “syndrome X”; and (2) “cataract” OR “lens opacity” OR “crystalline opacity” OR “lens opacities” OR “crystalline opacities”. The search was limited to research involving human subjects and included only studies published as full-text articles in English within peer-reviewed journals. Additionally, we manually reviewed the references of relevant original and review articles to identify further pertinent studies. The literature was assessed from the inception of the databases up to July 31, 2024.

### Inclusion and exclusion criteria

The inclusion criteria for potential studies were defined according to the PICOS framework:

P (Population): Adult population (aged 18 years or older).

I (Exposure): Subjects with MetS, which was diagnosed according to the criteria used in the primary studies.

C (Comparison): Subjects who did not meet the diagnostic criteria for MetS, as defined in the original studies. These individuals may have had one or more individual metabolic abnormalities but did not fulfil the full criteria for MetS diagnosis.

O (Outcome): Incidence or prevalence of cataract, comparing outcomes between those with and without MetS. The diagnosis of cataract was also consistent with the methods and criteria used in the primary studies.

S (Study Design): Observational studies, including cohort, case-control, and cross-sectional studies.

Exclusion criteria included reviews, editorials, meta-analyses, preclinical studies, studies that did not evaluate MetS as exposure, or studies that did not report the outcome of cataract. In cases of overlapping populations, the study with the largest sample size was selected for inclusion in the meta-analysis.

### Study quality evaluation and data extraction

The literature search, study identification, quality assessment, and data extraction were conducted independently by two authors, with any disagreements resolved by consulting the corresponding author. Study quality was evaluated using the Newcastle-Ottawa Scale (NOS) [31], which assesses selection, control of confounders, and outcome measurement and analysis, with scores ranging from 1 to 9, where 9 signifies the highest quality. Data collected for analysis included study details (author, year, country, and design), participant characteristics (source, sample size, age, and sex), the criteria for the diagnosis of MetS and number of subjects with MetS, follow-up periods for longitudinal studies, methods used to diagnose cataract, the number of cataract cases, and variables adjusted when analysing the association between MetS and cataract.

### Statistics

The association between MetS and cataract was analysed using risk ratios (RR) and 95% confidence intervals (CI), comparing between subjects with and without MetS. For studies that reported hazard ratios (HR), these were used directly as RR. For studies that provided odds ratios (OR), we converted these to RR using the formula: RR = OR/([1 − pRef] + [pRef × OR]), where pRef is the prevalence of the outcome in the reference group (participants without MetS) [32]. RR values and their standard errors were calculated from 95% CIs or *p*-values and logarithmically transformed for variance stabilization. To assess heterogeneity, we used the Cochrane Q test and I² statistics [33], with I² >50% indicating considerable heterogeneity. A random-effects model was applied to integrate the results, accounting for study variability [30]. Sensitivity analyses were conducted by excluding individual studies to test the robustness of the findings. Predefined subgroup analyses were performed to explore the effects of factors such as geographic region (Asian or Western countries), study design, average age, sex, diagnostic criteria for MetS, methods for confirmation of cataract cases, types of cataract (nuclear, cortical, and posterior capsular [PSC] cataract), and NOS scores. Subgroups were defined using the median values of continuous variables. Publication bias was evaluated using funnel plots and visual inspection for asymmetry, supplemented by Egger’s regression test [34]. Analyses were performed using RevMan (Version 5.1; Cochrane Collaboration, Oxford, UK) and Stata software (version 12.0; Stata Corporation, College Station, TX).

## Results

### Study inclusion

The study inclusion process is illustrated in Supplementary Fig. 1. Initially, 388 potentially relevant records were identified from the three databases, with 121 excluded due to duplication. A subsequent screening of titles and abstracts led to the exclusion of 246 studies, primarily because they did not align with the meta-analysis’s objectives. The full texts of the remaining 21 records were reviewed by two independent authors, resulting in the exclusion of 11 studies for reasons detailed in Supplementary Fig. 1. Ultimately, ten observational studies were deemed appropriate for the quantitative analysis [18–27].

### Overview of study characteristics

Table 1 presents the summarized characteristics of the included studies. Overall, four prospective cohort studies [19, 22, 24, 27], one case-control study [20], and five cross-sectional studies [18, 21, 23, 25, 26] were included. These studies were reported from 2007 to 2024, and conducted in Lithuania, Sweden, Italy, Singapore, Australia, Korea, Taiwan, and the United Kingdom. Community populations were included nine studies [18, 19, 21–27], and hospitalized patients for acute, non-neoplastic conditions were included in the other study [20]. Overall, 379464 subjects were included, and the mean ages of the patients were 49.9–63.9 years. The diagnosis of MetS was achieved via the IDF criteria in four studies [19, 20, 22, 24], and with the NCEP-ATP III criteria in the other six studies [18–21, 23, 25–27]. Accordingly, 81129 (21.4%) of the included participants had MetS. The follow-up durations of the prospective cohort studies were 8.2–15.0 years. The diagnosis of cataract was via ophthalmologic examination in five studies [18, 20–23], by International Classification of Disease (ICD) codes in three studies [19, 24, 27], and evidenced by self-reported clinically diagnosed cataract in two studies [25, 26]. In seven studies, cases of overall cataract were identified [18, 21–23, 25–27], while in the other three studies, only patients undergoing surgeries for cataract were identified as cases [19, 20, 24]. Accordingly, 44073 (11.6%) of the included participants had cataract. Multivariate analysis was performed in all of the included studies when the association between MetS and cataract was evaluated, with the adjustment of age, sex, and other potential confounding factors to a varying degree. The NOS scores of the included studies were seven to nine, suggesting overall good study quality (Supplementary Table 1).

**Table 1:** Summary of included studies.

Study	Country	Design	Participants	Sample size	Mean age (years)	Men (%)	Diagnostic criteria of MetS	No. of patients with MetS	Follow-up duration for cohort studies (years)	Methods for the diagnosis of cataract	Type of cataract	Number of patients with cataract	Variables adjusted
Paunksnis [18]	Lithuania	CS	Community people aged 35–64 years	1282	50	44.7	NCEP-ATP III	294	NA	Ophthalmologic examination	Overall cataract	236	Age and sex
Lindblad [19]	Sweden	PC	Community women aged 49–83 years	35,369	62.1	0	IDF	124	8.2	ICD-10 codes	Cataract surgery	4508	Age, smoking, alcohol consumption, use of steroid medication, use of vitamin supplement and educational level
Galeone [20]	Italy	CC	Hospitalized patients for acute, non-neoplastic conditions	2283	62	47	IDF	147	NA	Ophthalmologic examination	Cataract surgery	761	Age, sex, and study centre
Sabanayagam [21]	Singapore	CS	Community people aged 40–80 years	2794	58.2	48.3	NCEP-ATP III	1194	NA	Ophthalmologic examination	Overall cataract	1268	Age, sex, education, and smoking status
Maralani [22]	Australia	PC	Community people aged 35 years or older	1997	63.9	42.3	IDF	246	10	Ophthalmologic examination	Overall cataract	857	Age, sex, eye disease at baseline, preexisting DM, CAD, and stroke, and family history of blindness
Park [23]	Korea	CS	Community people aged 40 years or older	11,076	56	43.1	NCEP-ATP III	4364	NA	Ophthalmologic examination	Overall cataract	5406	Age, sex, income, education, residential area, smoking status, drinking alcohol, exercise, occupation, family history of eye disease, and sun exposure
Lindblad [24]	Sweden	PC	Community men aged 45–79 years	45,049	59.2	100	IDF	880	15	ICD-10 codes	Cataract surgery	7573	Age, smoking, alcohol consumption, steroid medication use, vitamin supplement use, educational level, and physical activity
Jee [25]	Korea	CS	Community people aged 50–77 years	40,262	57.9	47.7	NCEP-ATP III	6971	NA	Self-reported clinically diagnosed cataract	Overall cataract	1972	Age, sex, residence area, BMI, and energy intake
Chang [26]	Taiwan	CS	Community people aged 31–77 years	121,380	49.9	35.9	NCEP-ATP III	34,817	NA	Self-reported clinically diagnosed cataract	Overall cataract	17,320	Age, sex, smoking, alcohol drinking, exercise, HGB, TC, LDL-C, eGFR, and UA
Xu [27]	UK	PC	Community people aged 40–69 years	117,972	56.5	50	NCEP-ATP III	32,092	11	ICD-10 codes	Overall cataract	4172	Age, sex, ethnicity, education, SES, smoking, alcohol drinking, and sun exposure

*MetS* metabolic syndrome, *CS* cross-sectional, *PC* prospective cohort, *CC* case-control, *IDF* International Diabetes Federation, *NCEP-ATP III* National Cholesterol Education Program Adult Treatment Panel III, *NA* not applicable, *ICD-10* International Classification of Disease Tenth Edition, *DM* diabetes mellitus, *CAD* coronary artery disease, *HGB* haemoglobin, *TC* total cholesterol, *LDL-C* low-density lipoprotein cholesterol, *eGFR* estimated glomerular filtrating rate, *UA* uric acid, *SES* socioeconomic status.

### Results of meta-analysis and sensitivity analysis

**Fig. 1:**
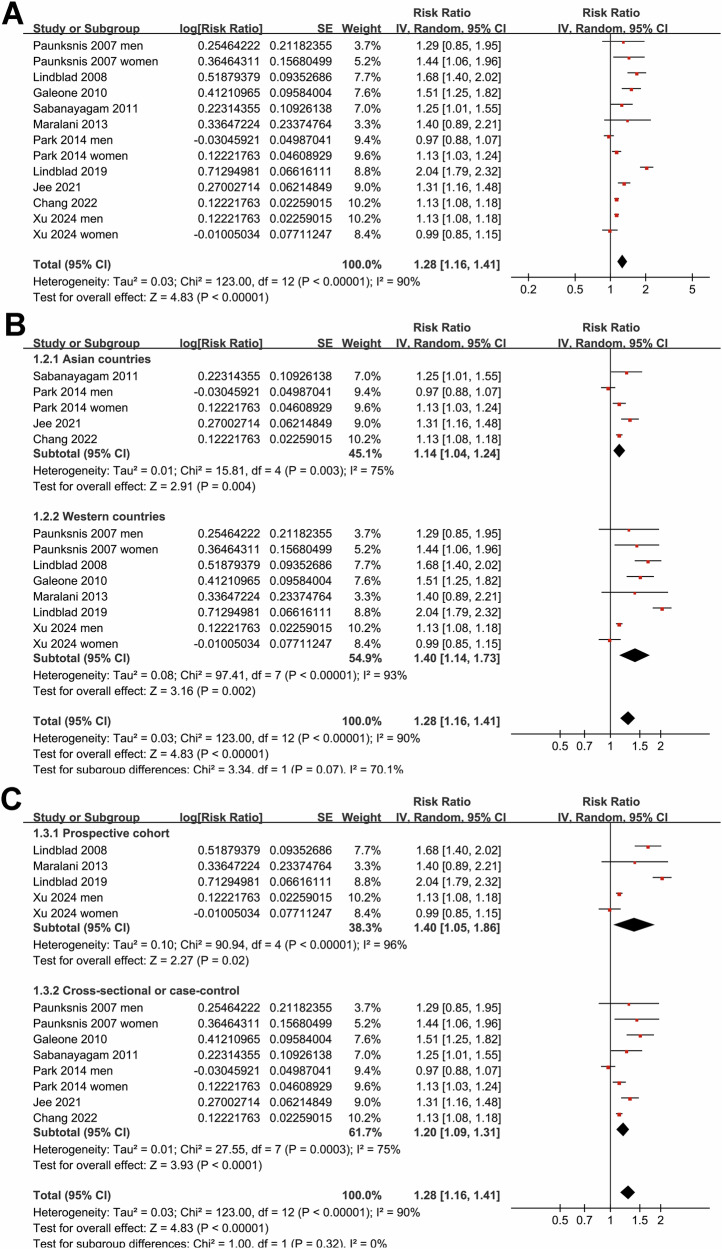
Forest plots representing the meta-analysis of the association between MetS and cataract in adult population. **A** Plots for the overall meta-analysis; **B** Plots for the subgroup analysis according to study country; and **C** Plots for the subgroup analysis according to study design.

Since three studies reported the outcome by gender separately [18, 23, 27], these datasets were included independently into the meta-analysis, making 13 datasets available for the overall meta-analysis. Overall, the pooled results of the 13 datasets from ten studies [18–27] showed that compared to subjects without MetS, adults with MetS were significantly associated with an increased risk of overall cataract (RR: 1.28, 95% CI: 1.16–1.41, *p* < 0.001; I^2^ = 90%; Fig. 1A). Sensitivity analysis by excluding one dataset at a time did not significantly change the results (RR: 1.25–1.32, *p* all <0.05).

### Results of the subgroup analyses

**Fig. 2:**
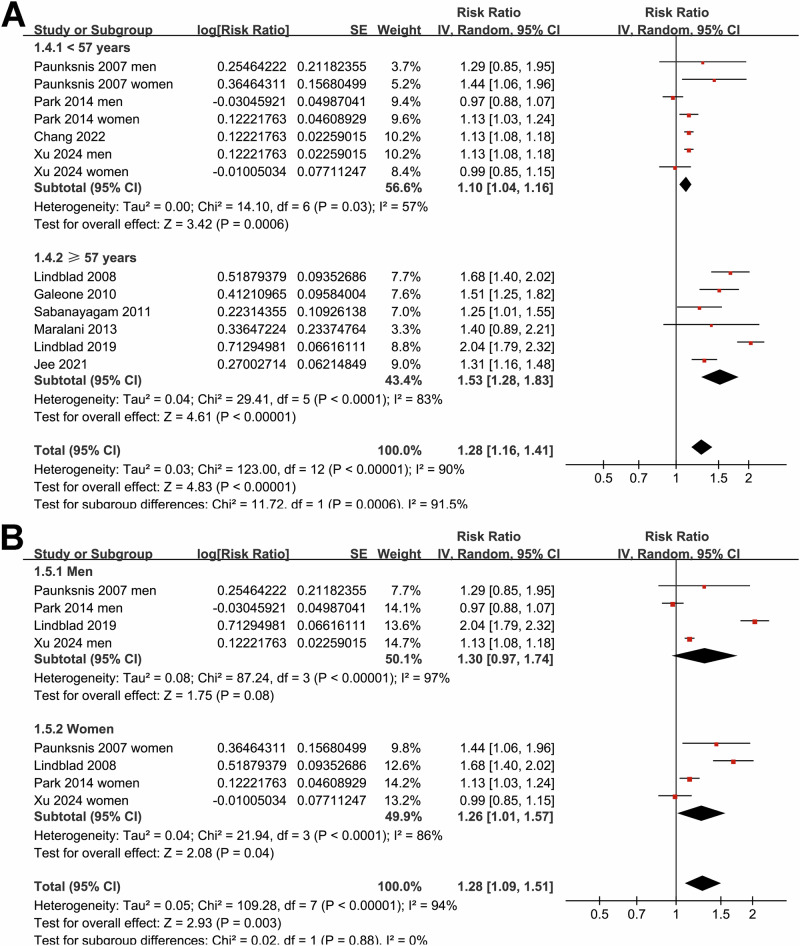
Forest plots representing the subgroup analyses of the association between MetS and cataract in adult population. **A** Subgroup analysis according to mean age of the participants; and **B** Subgroup analysis according to sex of the participants.

**Fig. 3:**
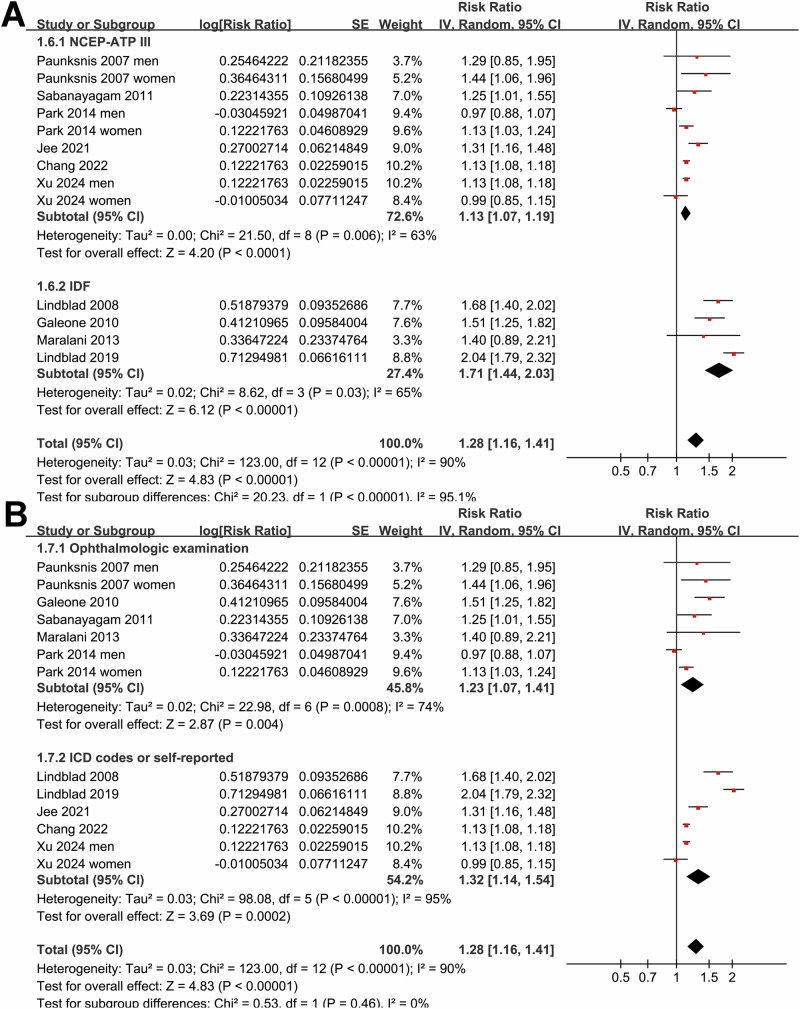
Forest plots representing the subgroup analyses of the association between MetS and cataract in adult population. **A** Subgroup analysis according to the diagnostic criteria for MetS; and **B** Subgroup analysis according to the methods for validation of cataract.

**Fig. 4:**
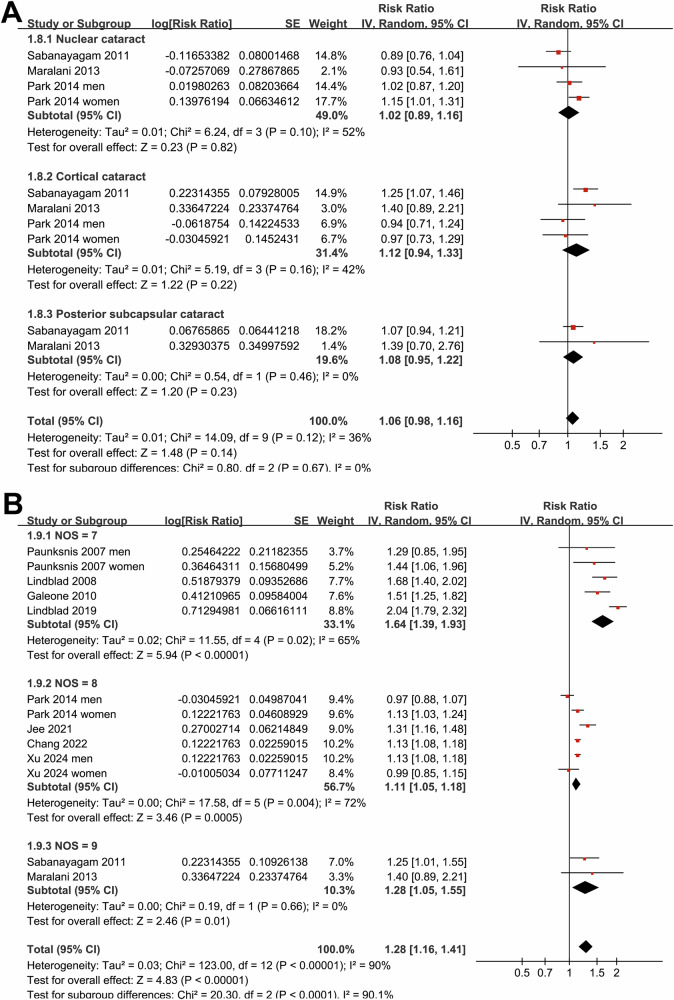
Forest plots representing the subgroup analyses of the association between MetS and cataract in adult population. **A** Subgroup analysis according to the type of cataract; and **B** Subgroup analysis according to NOS scores.

Subgroup analyses indicated that the association between MetS and cataract was not statistically different in studies from Western and Asian countries (*p* for subgroup difference = 0.07; Fig. 1B), or between prospective cohort and case-control/cross-sectional studies (*p* for subgroup difference = 0.32; Fig. 1C). Interestingly, a stronger association between MetS and cataract was observed in participants of mean age ≥57 years as compared to those <57 years (RR: 1.53 versus 1.10, *p* for subgroup difference <0.001; Fig. 2A), while a consistent association was observed in men and women (*p* for subgroup difference = 0.88; Fig. 2B). We also found that a stronger association between MetS and cataract in studies with MetS diagnosed by the IDF criteria compared to those with MetS diagnosed by the NCEP-ATP III criteria (RR: 1.71 versus 1.13, *p* for subgroup difference <0.001; Fig. 3A), while the association was similar between studies with cataract diagnosed by ophthalmologic examination and ICD codes/self-report (*p* for subgroup difference = 0.46; Fig. 3B). Furthermore, the subgroup analysis did not suggest a significant difference for the outcome of nuclear, cortical, or PSC cataract (*p* for subgroup difference = 0.67; Fig. 4A), while a stronger association was observed in studies with NOS score of seven, as compared to those of eight and nine (RR: 1.64 versus 1.11 and 1.28, *p* for subgroup difference < 0.001; Fig. 4B).

### Publication bias

Upon visual inspection, the funnel plots for meta-analysis of the association between MetS and cataract appear symmetrical, indicating a low likelihood of publication bias (Supplementary Fig. 2). Additionally, Egger’s regression test results (*p* = 0.44) also support this conclusion by suggesting a low risk of publication bias.

## Discussion

This meta-analysis of observational studies provides compelling evidence that MetS is associated with an increased risk of cataract in adults. By pooling data from ten studies comprising 379,464 participants, we found that individuals with MetS have a 28% higher risk of developing cataract compared to those without MetS. The results were consistent across sensitivity analyses, indicating the robustness of the findings. Subgroup analyses revealed that the association between MetS and cataract was significantly stronger in older populations (≥57 years) and when MetS was defined by the IDF criteria, suggesting potential age and diagnostic criteria-dependent effects on this association.

Several mechanisms may underlie the observed association between MetS and cataract development. MetS is a cluster of metabolic disturbances, including central obesity, hypertension, dyslipidaemia, and hyperglycaemia, each of which may independently or synergistically contribute to cataractogenesis [7]. Hyperglycaemia, a central component of MetS, is known to induce oxidative stress and osmotic damage in the lens [35]. Excess glucose in the aqueous humour can lead to the accumulation of sorbitol through the polyol pathway, resulting in osmotic stress that causes lens fibre swelling and protein aggregation, ultimately leading to lens opacity [15]. Additionally, oxidative stress from hyperglycaemia generates reactive oxygen species (ROS), which can damage lens proteins and lipid membranes [36].

Dyslipidaemia, characterized by elevated triglycerides and low high-density lipoprotein cholesterol (HDL-C), is another MetS component that may contribute to cataract formation [37]. High levels of serum lipids can lead to lipid peroxidation, generating cytotoxic aldehydes and ROS that can damage lens epithelial cells [38]. Furthermore, low HDL-C levels may impair the antioxidant capacity of the eye, further exacerbating oxidative damage [39]. Hypertension, another component of MetS, is associated with impaired ocular blood flow and increased oxidative stress, which may also contribute to cataract development [40]. Collectively, these pathological processes highlight the complex interplay between different components of MetS in cataractogenesis.

Our subgroup analyses provided further insights into the association between MetS and cataract. We found that the association was significantly stronger in older adults (≥57 years) than in younger participants. This finding is consistent with the notion that aging is a significant risk factor for cataract [41] and suggests that MetS may have an additive effect on cataract development in older individuals. Aging is associated with a decline in the body’s antioxidant defence mechanisms and an increase in oxidative damage [42], which can compound the effects of MetS components such as hyperglycaemia, dyslipidaemia, and hypertension. Thus, the metabolic and oxidative stress induced by MetS may accelerate cataract formation in older individuals.

Interestingly, our subgroup analysis showed that the association between MetS and cataract was stronger in studies using the IDF criteria compared to those using the NCEP-ATP III criteria. This difference may be explained by the structural distinctions between the two diagnostic definitions. The IDF definition mandates the presence of central obesity—assessed by waist circumference thresholds adjusted for ethnicity—plus at least two additional metabolic abnormalities, while the NCEP-ATP III criteria require any three out of five components, allowing central obesity to be absent [43, 44]. Central obesity plays a pivotal role in the pathophysiology of MetS by promoting systemic inflammation, oxidative stress, and adipokine dysregulation, all of which can accelerate lens protein aggregation and cataract formation [45]. As such, the IDF criteria may capture individuals with a more adiposity-driven metabolic phenotype and a higher oxidative burden. This may partly explain the stronger observed association with cataract, emphasizing the potential importance of central obesity in cataractogenesis beyond the contribution of other MetS components. On the other hand, the NCEP-ATP III criteria allow for more variability in MetS diagnosis without central obesity, potentially diluting the association between MetS and cataract risk [46]. However, the underlying reasons for the observed variation in effect size remain unclear, and no definitive mechanistic explanation can be drawn at this stage. Further studies are warranted to clarify whether the choice of MetS definition influences the strength of its association with cataract risk.

This meta-analysis has several strengths. It may be the most up-to-date meta-analysis that quantitatively assesses the association between MetS and cataract risk. We conducted a thorough literature search across multiple databases, included a large sample size, and performed rigorous subgroup and sensitivity analyses. Our use of a random-effects model accounted for variability among studies, and our assessments of publication bias suggested a low likelihood of such bias affecting our results. Furthermore, the inclusion of both prospective and retrospective studies provides a broad perspective on the association across different study designs. However, several limitations should be considered when interpreting our findings. First, substantial heterogeneity was observed across the included studies, likely due to differences in study design, population characteristics, diagnostic criteria for MetS and cataract, and adjustments for confounding factors. While we conducted subgroup analyses to explore potential sources of heterogeneity, not all variability could be accounted for. Second, most included studies were observational, which limits the ability to infer causality. Residual confounding from unmeasured factors, such as genetic predisposition, dietary habits, and other comorbidities, may also affect the observed associations. Additionally, in most included studies, control groups consisted of participants who did not meet the criteria for MetS but may have had one or more metabolic abnormalities. This heterogeneity in the comparator group could influence the observed effect estimates and should be considered when interpreting the results. Finally, the reliance on self-reported cataract diagnoses in some studies may introduce misclassification bias, potentially underestimating or overestimating the true association. However, the subgroup analysis showed consistent results in studies with cataract validated by ophthalmologic examination and by ICD codes/self-reported diagnosis.

The findings of this meta-analysis have important clinical implications. Given the rising prevalence of MetS worldwide, our results suggest that individuals with MetS should be considered at higher risk for cataract development, particularly as they age. Clinicians should be aware of this association and consider regular ophthalmologic assessments for patients with MetS to facilitate early detection and management of cataract. Lifestyle modifications and interventions targeting MetS components, such as weight management, blood pressure control, lipid-lowering therapy, and glycaemic control, may not only reduce cardiovascular risk but also potentially decrease the risk of cataract formation [47]. Future research should aim to address the limitations of the current evidence base. High-quality prospective cohort studies with standardized diagnostic criteria for MetS and cataract, adequate adjustment for confounding factors, and consideration of potential effect modifiers (such as age, sex, and race) are needed to strengthen the evidence for a causal relationship. Investigating the individual and combined effects of MetS components on cataract risk would also provide valuable insights into the underlying mechanisms. Moreover, research on the role of novel biomarkers and genetic factors in the MetS-cataract link could help identify high-risk populations and inform personalized prevention strategies [48]. Finally, although our subgroup analysis indicated a stronger association between MetS and cataract among older individuals (≥57 years), the increasing prevalence of MetS at younger ages raises concerns about its potential role in accelerating age-related ocular changes earlier in life [49]. Prolonged exposure to metabolic abnormalities—such as hyperglycaemia, hypertension, and dyslipidaemia—beginning in early adulthood may contribute to earlier lens aging, although current data on early-onset cataract remain limited. Future studies focusing on younger populations with MetS are warranted to evaluate this possibility. Early intervention to manage MetS components may be beneficial not only for systemic health but also for reducing long-term ocular risks, including cataractogenesis.

## Conclusions

In conclusion, this meta-analysis suggests a significant association between MetS and an increased risk of cataract in adults, with stronger effects observed in older populations and when MetS is defined by IDF criteria. The findings underscore the potential importance of metabolic health in cataract prevention and highlight the need for comprehensive management of MetS components to mitigate cataract risk. Further research is warranted to clarify the underlying mechanisms, confirm causality, and explore the impact of targeted interventions on cataract prevention in individuals with MetS.

## Summary

### What was known before

- Metabolic syndrome (MetS) has been suggested to increase the risk of cataracts, but the evidence from individual studies has been inconsistent.

### What this study adds

- This meta-analysis of 10 studies involving 379,464 participants confirms that MetS is associated with a 28% increased risk of cataract.
- The association between MetS and cataract risk is stronger in individuals aged ≥57 years and in studies using the International Diabetes Federation criteria for MetS diagnosis.
- These findings highlight the importance of targeted prevention strategies for older populations with MetS to reduce the risk of cataracts.

## Supplementary information

Supplemental materials

## Data Availability

The datasets generated during and analysed during the current study are not publicly available due to the need for further research but are available from the corresponding author on reasonable request.
