# Supplementary material for: Association between metabolic syndrome and cataract: a meta-analysis
Source: Eye (Lond). 2025 Jul 11;39(13):2555–64. doi: 10.1038/s41433-025-03910-2 (PMC12402076; doi:10.1038/s41433-025-03910-2)
Supplement: Supplementary file 1 — Supplemental materials [file 41433_2025_3910_MOESM1_ESM.docx]

**Supplemental Figure 1** Flowchart of database search and study inclusion;


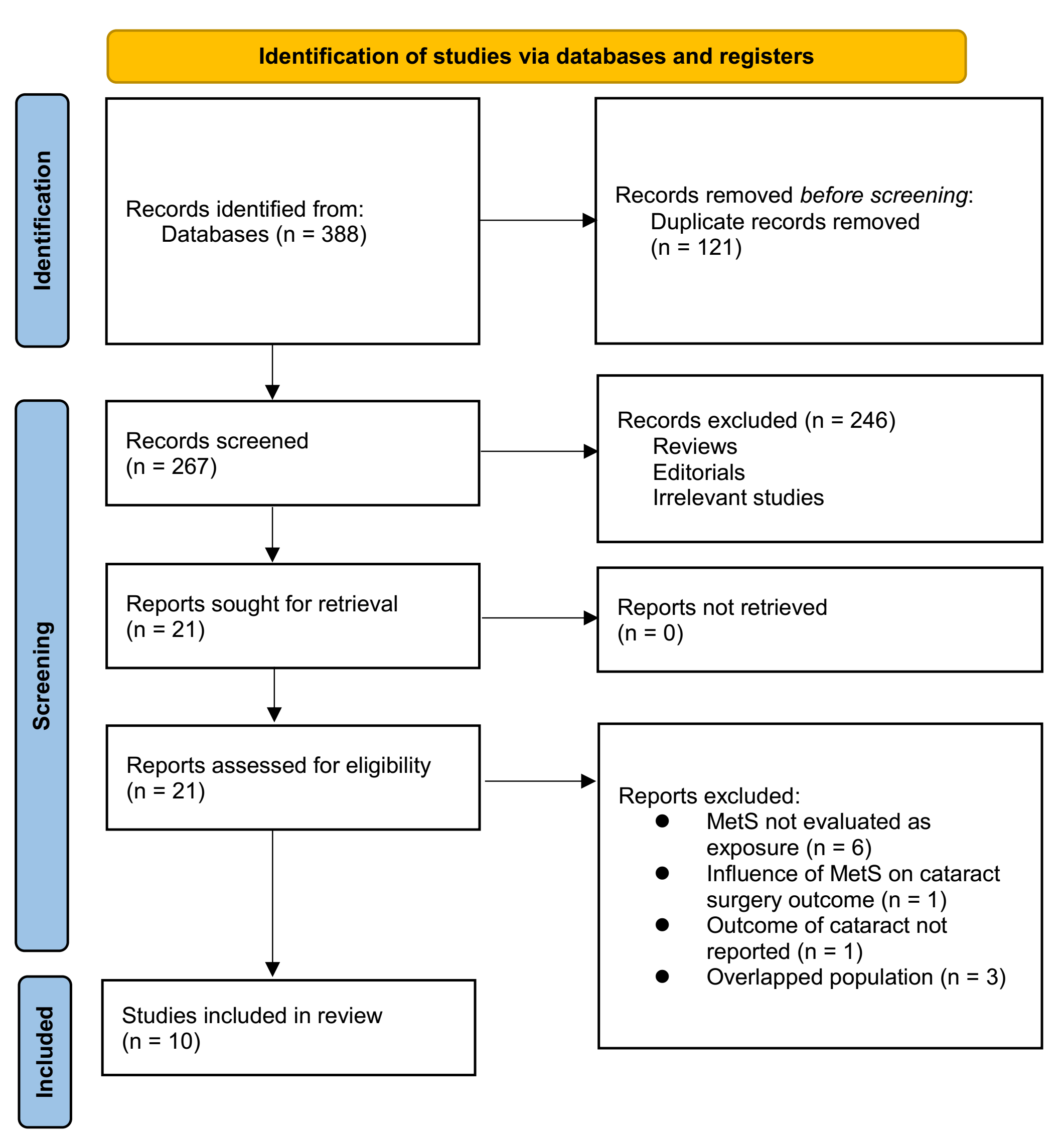


**Supplemental Figure 2** Funnel plots for the meta-analysis of the association between MetS and cataract in adult population;


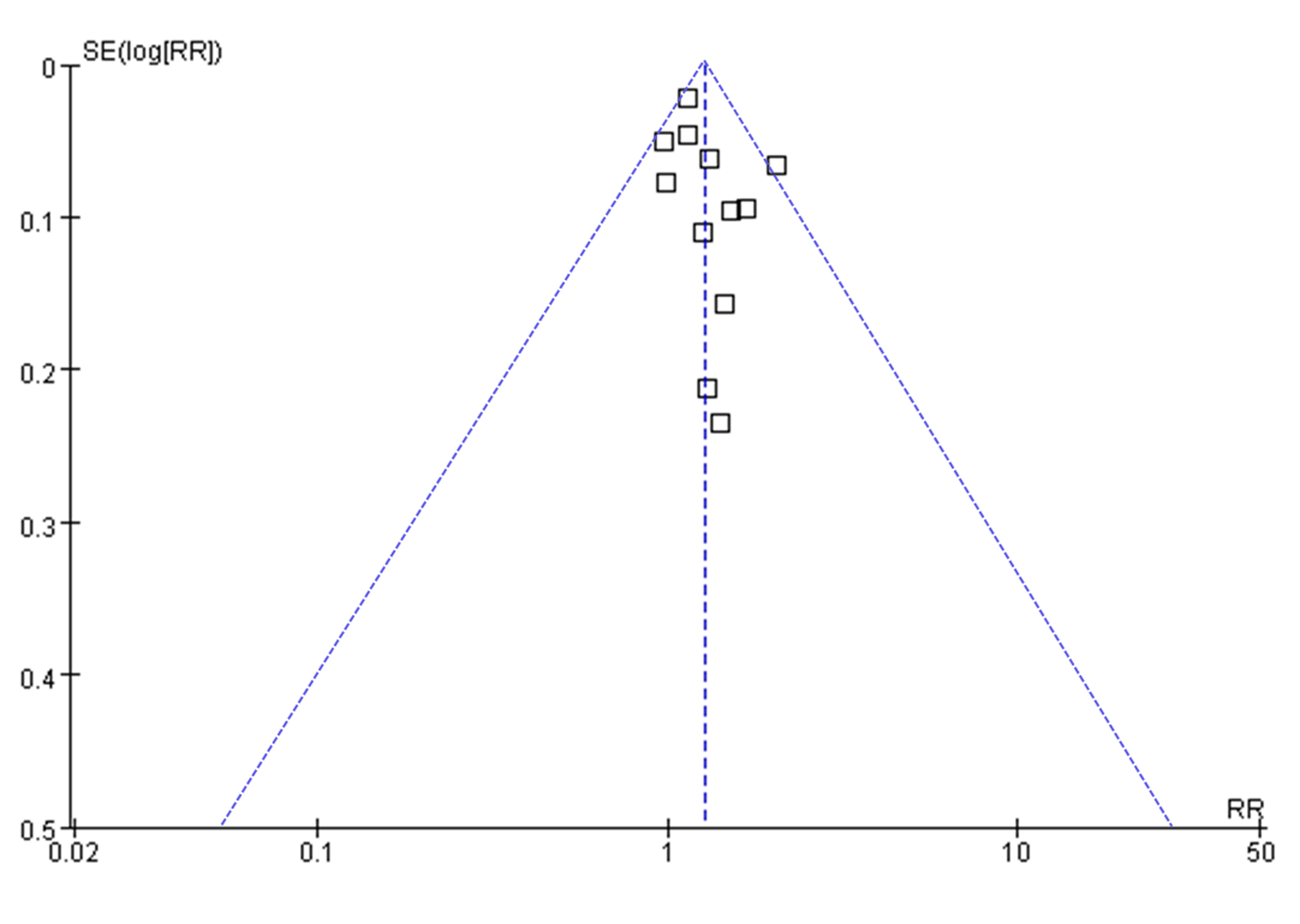


**Supplemental Table 1** Study quality evaluation via the Newcastle-Ottawa Scale

| Cohort study | Representativeness of the exposed cohort | Selection of the non-exposed cohort | Ascertainment of exposure | Outcome not present at baseline | Control for age and sex | Control for other confounding factors | Assessment of outcome | Enough long follow-up duration | Adequacy of follow-up of cohorts | Total |
| --- | --- | --- | --- | --- | --- | --- | --- | --- | --- | --- |
| Lindblad 2008 | 1 | 1 | 0 | 1 | 1 | 1 | 0 | 1 | 1 | 7 |
| Maralani 2013 | 1 | 1 | 1 | 1 | 1 | 1 | 1 | 1 | 1 | 9 |
| Lindblad 2019 | 1 | 1 | 0 | 1 | 1 | 1 | 0 | 1 | 1 | 7 |
| Xu 2024 | 1 | 1 | 1 | 1 | 1 | 1 | 0 | 1 | 1 | 8 |
|  | | | | | | | | | | |
| Cross-sectional or case control study | Adequate definition of cases | Representativeness of cases | Selection of controls | Definition of controls | Control for age and sex | Control for other confounders | Exposure ascertainment | Same methods for events ascertainment | Non-response rates | Total |
| Paunksnis 2007 | 1 | 0 | 1 | 1 | 1 | 0 | 1 | 1 | 1 | 7 |
| Galeone 2010 | 1 | 0 | 1 | 1 | 1 | 0 | 1 | 1 | 1 | 7 |
| Sabanayagam 2011 | 1 | 1 | 1 | 1 | 1 | 1 | 1 | 1 | 1 | 9 |
| Park 2014 | 1 | 0 | 1 | 1 | 1 | 1 | 1 | 1 | 1 | 8 |
| Jee 2021 | 1 | 0 | 1 | 1 | 1 | 1 | 1 | 1 | 1 | 8 |
| Chang 2022 | 1 | 0 | 1 | 1 | 1 | 1 | 1 | 1 | 1 | 8 |
